# Impact of CT-relevant Skeletal Muscle Parameters on Post-chemotherapy Survival in Patients with Unresectable Pancreatic Ductal Adenocarcinoma

**DOI:** 10.2174/0115734056356822250205174104

**Published:** 2025-02-17

**Authors:** Siying Zhang, Zhenping Wu, Guo Sa, Zhan Feng, Feng Chen

**Affiliations:** 1 Department of Radiology, The First Affiliated Hospital, School of Medicine, Zhejiang University, Hangzhou 310003, Zhejiang, China; 2 Department of Critical Care Medicine, The First Affiliated Hospital, School of Medicine, Zhejiang University, Hangzhou 310003, Zhejiang, China

**Keywords:** Sarcopenia, Myosteatosis, Chemotherapy, Pancreatic adenocarcinoma, Survival, CT

## Abstract

**Purpose:**

The study aimed to investigate the association of CT-relevant skeletal muscle parameters, such as sarcopenia and myosteatosis, with survival outcomes in patients receiving chemotherapy for unresectable pancreatic ductal adenocarcinoma (PDAC).

**Methods:**

In this retrospective analysis, patients who began chemotherapy for unresectable PDAC were included. Sarcopenia and myosteatosis were assessed on pretreatment CT at the L3 level by skeletal muscle index and mean muscle attenuation with predefined cutoff values. The Cox proportional hazards model was used to analyze the factors associated with progression-free survival (PFS) and overall survival (OS).

**Results:**

A total of 150 patients were enrolled. Compared to patients without sarcopenia, patients with sarcopenia had significantly worse PFS (p=0.003) and OS (p<0.001). Patients with myosteatosis had significantly worse PFS (p=0.01) and OS (p=0.002) compared to those without myosteatosis. In multivariate analysis, after adjusting for age, sex, tumor size, location, treatment modality, smoking, drinking, underlying diseases, and partial laboratory tests, sarcopenia remained an independent predictor of PFS (p=0.006) and OS (p<0.001). Myosteatosis remained an independent predictor of OS (p=0.008), but not of PFS.

**Conclusion:**

Sarcopenia and myosteatosis are independent prognostic factors for patients with unresectable pancreatic ductal adenocarcinoma after chemotherapy.

## 1 INTRODUCTION

Pancreatic ductal adenocarcinoma (PDAC) is one of the deadliest malignant tumors, with the majority of patients being diagnosed in an unresectable state and a 5-year survival rate below 10% [1]. Despite great advancements in standard first-line chemotherapy regimens, such as combining gemcitabine with nab-paclitaxel (AG) and modified folfirinox (mFFX), the prognosis for unresectable PDAC remains concerning [2]. The side effects of the treatment may lead to a reduced quality of life [3]. Consequently, the identification of novel prognostic factors is essential for improving the management and prognosis of patients with unresectable PDAC.

Sarcopenia is a condition defined by muscular debility and a decline in physical function caused by a reduction in skeletal muscle mass [4, 5]. It has adverse effects on body composition as well as immune function. For patients with unresectable PDAC receiving chemotherapy, muscle atrophy before treatment initiation or changes during treatment have been associated with poor prognosis [6-8]. For patients undergoing chemotherapy or immunotherapy for other malignancies, such as renal, liver, gastric, and lung cancers, muscle atrophy has been linked to a negative prognosis and suboptimal treatment response [9-14]. It is also important to investigate other specific conditions related to sarcopenia. Patients might experience concurrent loss of skeletal muscle and an increase in adipose tissue, resulting in a condition called sarcopenic obesity [15, 16]. This condition has been linked to poorer progression-free survival (PFS) in patients with malignant melanoma undergoing immunotherapy [17]. Sarcopenic obesity is similarly linked to poorer overall survival (OS) in hepatocellular carcinoma (HCC) patients undergoing immunotherapy [9]. Additionally, muscle wasting can be paired with a rise in intermuscular and intramuscular fat, a condition known as myosteatosis [18]. Myosteatosis becomes more prevalent with increasing age and obesity and is associated with metabolic dysfunction and reduced strength and mobility. This condition is also associated with reduced OS in patients with various cancers [19, 20].

The evaluation of sarcopenia, myosteatosis, and sarcopenic obesity can be conducted using computed tomography (CT) images at the level of the third lumbar vertebra (L3) [18-20]. Currently, there is limited research on the relationship among sarcopenia, myosteatosis, and sarcopenic obesity, and the prognosis of patients with unresectable PDAC who are undergoing chemotherapy. This study aimed to investigate whether these muscle-related parameters could be used to predict treatment outcomes in patients with unresectable PDAC receiving chemotherapy.

## 2 PARTICIPANTS AND METHODS

### 2.1 Study Population

We included patients diagnosed with unresectable PDAC who received chemotherapy including AG and mFFX at our hospital between March 2020 and February 2023.

Information was extracted from our hospital’s (the First Affiliated Hospital, Zhejiang University School of Medicine) electronic records. Our study received approval from the clinical research ethics committee of the First Affiliated Hospital, Zhejiang University School of Medicine, with approval number IIT20241570A.

Pre-treatment data, including age, sex, height, weight, tumor size, location, stage according to the 8th edition of AJCC, treatment modality, smoking and alcohol history, the presence of comorbidities, such as solid tumors, diabetes, hypertension, coronary heart disease, underlying lung diseases, and hepatitis B, as well as laboratory parameters, such as complete blood count, liver and kidney function, tumor markers, and treatment response, were collected from medical records. Informed consent was waived due to the retrospective nature of this study.

Contrast-enhanced CT scans were performed within one month prior to treatment utilizing portal venous phase scans. CT scans were performed using a 64-slice spiral CT scanner (Revolution EVO, General Electric Medical Systems). Tumor assessment was done following the response evaluation criteria in solid tumors (RECIST) 1.1 [21]. Objective tumor response was defined as the percentage of patients who achieved either a complete or partial response to treatment according to RECIST 1.1 criteria. Disease control was defined as the percentage of patients who achieved complete response, partial response, or stable disease as per RECIST 1.1 criteria. Patients were excluded from the study if pre-treatment CT scan images and clinical data were not available. All patients were followed up until February 14, 2024.

### 2.2 CT-relevant Skeletal Muscle Parameters Evaluation

An experienced abdominal radiologist with 15 years of expertise in abdominal imaging performed the evaluation of sarcopenia, myosteatosis, and sarcopenic obesity. This radiologist, who was blinded to the clinical outcomes, delineated all patients on axial portal-venous phase CT images using 3DSlicer (v. 4.10.2, www.slicer.org). The cross-sectional area of skeletal muscle at the level of the third lumbar spine was assessed. Skeletal muscle was identified and quantified using Hounsfield unit (HU) thresholds of -29 to +150 [9, 22].

**Fig. (1):**
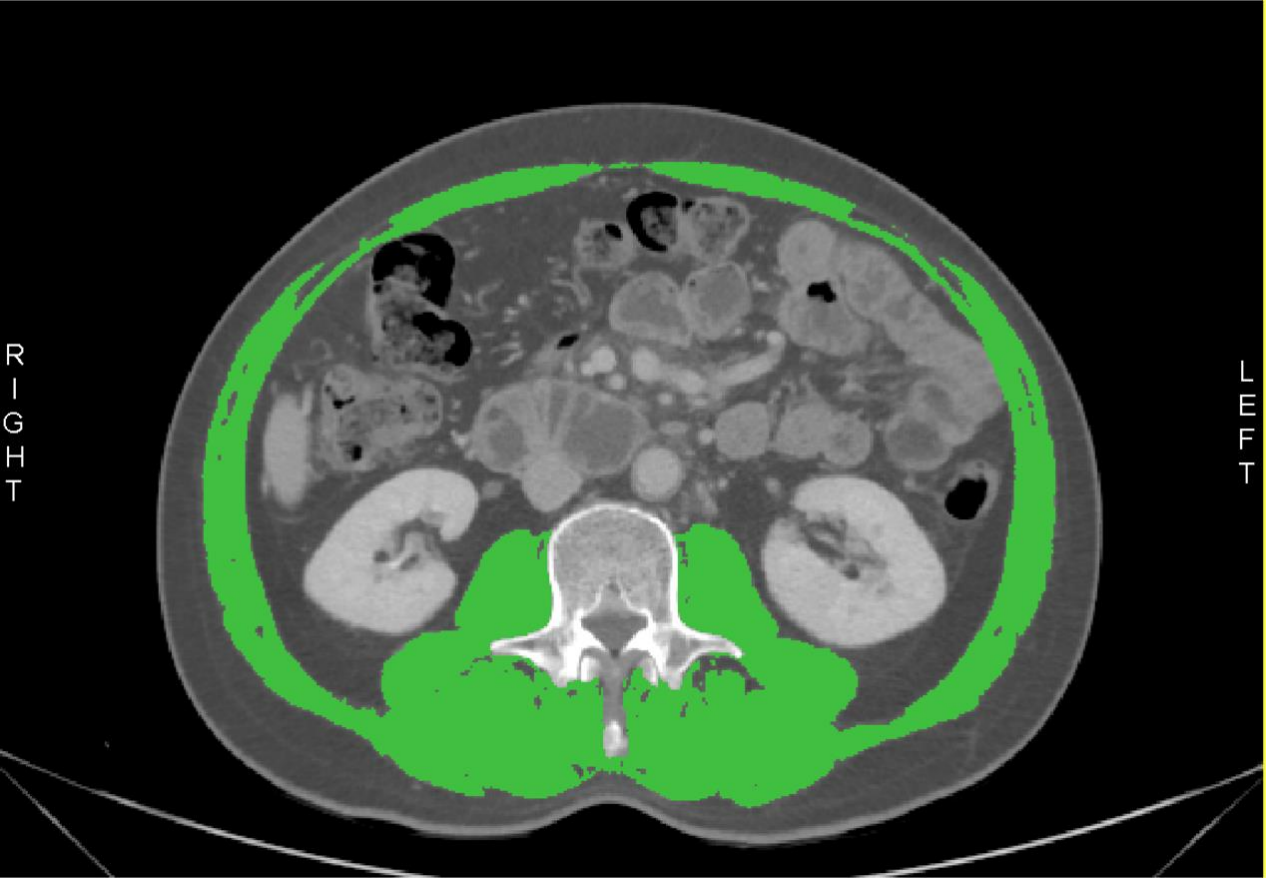
Skeletal muscle assessment using computed tomography.Muscle segmentation was conducted on the cross-sectional image at the L3 vertebra, using a threshold range of −29 to +150 HU (shown in green). This included the psoas major, erector spinae, quadratus lumborum, transversus abdominus, as well as the external and internal oblique muscles, and the rectus abdominus.

The muscles in the L3 region included the psoas, erector spinae, quadratus lumborum, transversus abdominis, external and internal obliques, and rectus abdominis. The cross-sectional area (CSA, cm2) was automatically computed by summing tissue pixels and multiplying by the pixel surface area (Fig. **1**). The skeletal muscle index (SMI) was calculated by dividing the total cross-sectional area (cm^2^) of skeletal muscle at L3 level by the square of the individual's height (m^2^). Cutoff values of 40.8 cm2/m^2^ for men and 34.9 cm2/m^2^ for women, commonly used in Asian countries for patients with gastrointestinal cancers, were applied to define sarcopenia [9, 23-25]. Muscle density reflecting fat infiltration was assessed as mean skeletal muscle density (SMD) in HU of the whole muscle region of the third lumbar spine. Myosteatosis was defined using mean SMD cutoff values of < 41 HU for patients with a body mass index (BMI) < 25 kg/m^2^ and < 33 HU for those with a BMI ≥ 25 kg/m^2^ [9, 19]. Sarcopenic obesity was identified in patients who exhibited both sarcopenia and either overweight or obesity (BMI > 25 kg/m^2^) [25].

### 2.3 Statistical Analysis

Data were expressed as mean ± standard deviation. Comparisons of patient characteristics between those with and without sarcopenia and myosteatosis were performed using Pearson chi-square or Fisher’s exact test for categorical variables and Student’s t-test or Mann–Whitney U test for continuous variables, depending on the distributional properties of each variable. PFS and OS were calculated from the start date of treatment to the event date or last follow-up. Survival outcomes were estimated using the Kaplan–Meier method, and associations with potential predictors of PFS and OS were evaluated through log-rank tests and univariate analysis. A Cox proportional hazards model was used for multivariate analysis to investigate potential predictors of survival outcomes, utilizing a stepwise variable selection. A significance level of <0.05 (two-sided) was considered statistically significant. Statistical analyses were performed using R version 4.0.2 (R Foundation for Statistical Computing). Nomogram multivariate logistic regression analysis was performed to create a model that predicts 3-month PFS and 12-month OS. A nomogram was constructed using selected covariates with the R statistical package rms (R Foundation for Statistical Computing) to improve interpretability.

## 3 RESULTS

### 3.1 Patient Characteristics

A total of 150 patients participated in the study from March 2020 to February 2023. Their demographic, clinical, and imaging characteristics are shown in Table **1**. The number of male patients was approximately twice that of female patients. Most tumors had a diameter exceeding 3 centimeters and were distributed similarly in the pancreatic head, neck, and body-tail regions. Tumor staging predominantly indicated stages 3 and 4 according to the AJCC 8th edition [26]. A minority of patients had other solid tumors, diabetes, coronary artery disease, underlying lung diseases, or hepatitis B. Additionally, a small number of patients had a history of smoking or alcohol consumption at the initiation of chemotherapy/immunotherapy combined treatment. Treatment modalities included AG, mFFX, AG combined with immunotherapy, and mFFX combined with immunotherapy.

The median follow-up duration was 28.2 months (95% CI, 26.37-31.65 months). The objective tumor response rate was 68.34%, with a disease control rate of 94.01%. The median PFS and OS were 8.09 months (95% CI, 6.52-9.87) and 12.5 months (95% CI, 11.30-14.67), respectively. The average values for BMI, SMI, SMD, and CSA across the entire cohort were 21.54 kg/m^2^, 40.91 cm2, 43.08 HU, and 111.33 cm2, respectively. Sarcopenia was identified in 57 (38%) patients, and myosteatosis was present in 39 (26.5%) patients. None of the patients showed sarcopenic obesity.

### 3.2 Comparison of Variables in Patients with and without Sarcopenia and Myosteatosis

Compared to patients without sarcopenia, those with sarcopenia had a reduced likelihood of having hypertension (p=0.013) and were less likely to achieve disease control (p=0.011) Table **1**. Patients with sarcopenia had lower BMI (p=0.012), SMI (p<0.001), SMD (p=0.017), and CSA (p<0.001), than those without sarcopenia. Compared to patients without myosteatosis, those with myosteatosis had a higher proportion of females (p<0.01), fewer tumors with a maximum diameter >3.0 cm (p=0.047), and were more likely to have concomitant solid tumors (p=0.017). Patients with myosteatosis had lower SMI (p=0.005), SMD (p<0.001), and CSA (p=0.006) than those without myosteatosis.

### 3.3 Associations of Sarcopenia and Myosteatosis with PFS and OS

**Fig. (2):**
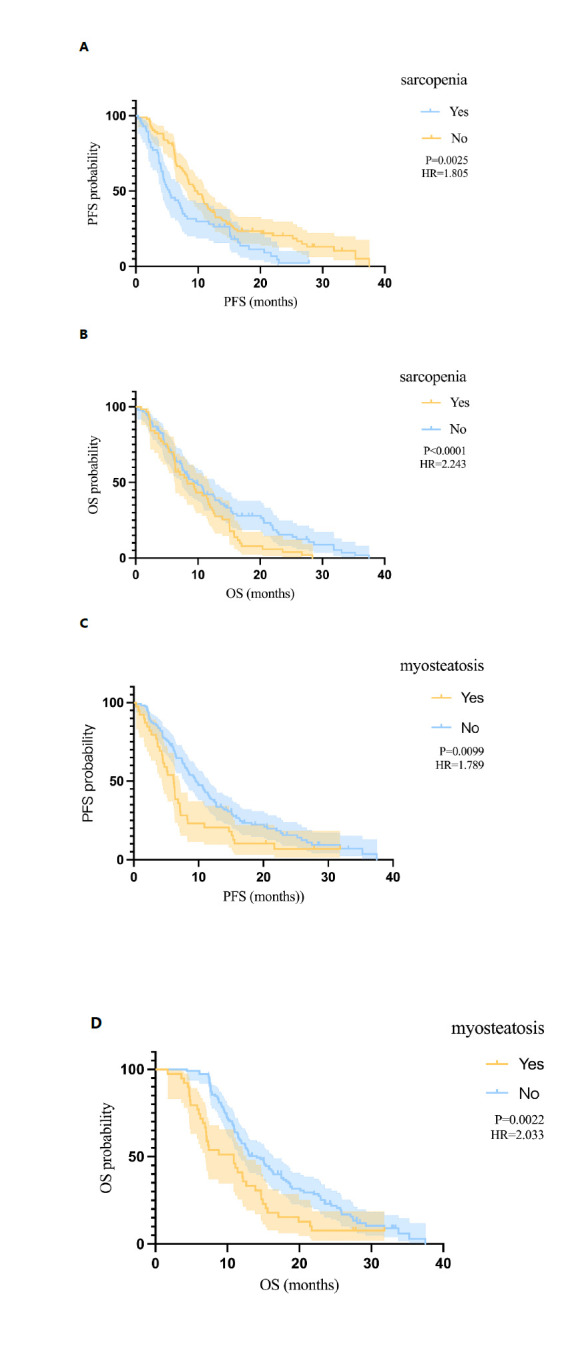
Kaplan−Meier curves of (**A**) PFS according to sarcopenia and (**B**) OS according to sarcopenia; (**C**) PFS according to myosteatosis and (**D**) OS according to myosteatosis. p values were calculated using the log-rank test.

Patients with sarcopenia had significantly worse PFS (median, 5.37 *vs.* 9.87 months, p=0.003; (Fig. **2A**) and OS (median, 9.67 *vs.* 14.67 months, p<0.001; (Fig. **2B**) compared to those without sarcopenia. Analogously, patients with myosteatosis had significantly worse PFS (median, 6.17 *vs.* 9.67 months, p=0.01; (Fig. **2C**) and OS (median, 10.87 *vs.* 14.07 months, p=0.002; (Fig. **2D**).

Univariate and multivariate analyses for PFS and OS are shown in Table **2**.

In multivariate analysis, after adjusting for age, sex, tumor size, location, treatment modality, smoking, drinking, underlying diseases, and partial laboratory tests, sarcopenia remained an independent predictor for both PFS (HR = 1.699, 95% CI: 1.165-2.478, p=0.006) and OS (HR = 2.023, 95% CI: 1.394-2.934, p<0.001). Myosteatosis served as an independent predictor for OS (HR = 1.735, 95% CI: 1.154-2.608, p=0.008), but not for PFS (Table **2**).

### 3.4 Nomogram and Prediction Model for 3-month PFS and 12-month OS

**Fig. (3):**
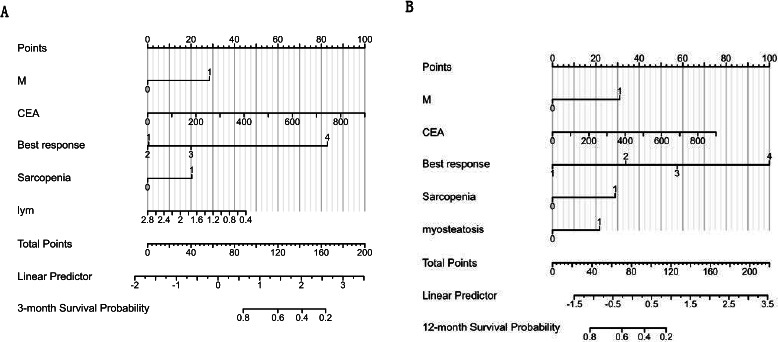
Nomograms predicting (**A**) 3-month PFS and (**B**) 12-month OS.

Through the stepwise variable selection within the multivariate analysis, we identified the optimal model for predicting PFS. Variables, such as M, CEA, lymphocytes, best response, and sarcopenia emerged as significant predictors of PFS. This model was used as a foundation to construct a nomogram for estimating the probability of 3-month PFS (c index: 0.70, 95% CI: 0.67–0.72; (Fig. **3A**). Every variable was attributed a score on the points scale. By aggregating and placing the scores on the total score scale, the likelihood of achieving 3-month PFS could be estimated.

In addition to sarcopenia and myosteatosis, M, CEA, and best response were also predictors for OS. Utilizing these variables, we devised a nomogram to forecast 12-month OS (c index: 0.69, 95% CI: 0.67–0.72; (Fig. **3B**).

## 4 DISCUSSION

Our investigation revealed the presence of skeletal muscle abnormalities in individuals diagnosed with unresectable PDAC; both sarcopenia and myosteatosis correlated with diminished PFS and OS. After accounting for variables, such as patient demographics, tumor extent, treatment modality, and selected laboratory parameters, sarcopenia stood as an independent prognostic factor for both PFS and OS. However, myosteatosis served as an independent predictor only for OS and not PFS. The study did not identify any cases of sarcopenic obesity. Unresectable PDAC is typically diagnosed in advanced stages, where patients are more prone to cancer cachexia, leading to the depletion of both muscle and fat tissues. In such circumstances, sarcopenic obesity may be relatively uncommon.

Muscle wasting is a primary characteristic of cancer cachexia [27, 28]. Our study found that patients with sarcopenia had a lower BMI compared to non-sarcopenic patients, which may be related to cancer cachexia. Cancer-related sarcopenia is linked to increased incidence rates, elevated mortality, heightened treatment-related toxicities, extended hospitalizations, reduced adherence to anticancer therapy, and a decline in quality of life [29].

Our research indicated sarcopenia to be a negative prognostic factor for patients with unresectable PDAC undergoing chemotherapy or chemotherapy combined with immunotherapy, consistent with previous studies. Previous research has indicated sarcopenia to serve as an independent predictor of poor prognosis in patients with PDAC undergoing chemotherapy, as found by Tomoya *et al*. [2]. Similarly, Hiroyuki *et al*. [30] also identified sarcopenia as a prognostic factor in elderly PDAC patients receiving chemotherapy. Similarly, the study by Bahattin *et al*. [8] suggested that patients diagnosed with sarcopenia may exhibit poorer overall survival rates in pancreatic cancer. Additionally, Jin *et al*.'s findings demonstrated a correlation between sarcopenia and lower OS and PFS [31].

With the progression of research on sarcopenia, myosteatosis has been identified as a distinct pathological condition entity separate from sarcopenia [18]. Studies have demonstrated that myosteatosis does not necessarily coexist with sarcopenia, can be influenced by varying clinical factors [32], and may hold independent prognostic value [33-35]. For example, Youn *et al*. [36] showed that low SMD serves as a predictor of poorer OS in patients treated with nivolumab for metastatic melanoma. Similarly, Chen *et al*. [9] found myosteatosis is an independent prognostic factor in patients undergoing immunotherapy for advanced HCC. Our findings indicated myosteatosis to be associated with a lower disease control rate and poor OS, although it did not correlate with PFS. The reduced muscle quality indicated by myosteatosis may reflect underlying impairment in host immune defense mechanisms, leading to decreased responsiveness to immunotherapy and worse clinical outcomes [37].

In general, reduced lymphocyte levels serve as an adverse prognostic indicator for pancreatic cancer, potentially indicating immune suppression and heightened tumor invasiveness [38]. Compromised immune function may render patients more susceptible to infections and other complications, exacerbating their condition and leading to a poorer prognosis. In numerous cancer types, the lymphocyte-to-monocyte ratio (LMR) and platelet-to-lymphocyte ratio (PLR) are recognized as pivotal prognostic factors [39-41]. However, our findings have indicated a lack of correlation between LMR and PLR and overall survival in patients with unresectable PDAC undergoing chemotherapy or chemotherapy combined with immunotherapy, consistent with previous studies [42, 43]. Furthermore, we observed CEA to independently predict both PFS and OS, whereas CA125 and CA19-9 did not. This inconsistency with prior research could be attributed to our limited sample size and the absence of dynamic monitoring of these biomarkers [44].

Establishing a survival prediction model helped effectively forecast the survival period and disease progression in unresectable PDAC patients. A nomogram developed from five variables (M, CEA, best response, sarcopenia, and lymphocyte count) provided reliable prognostic estimates for our patients' 3-month PFS (C-index: 0.70), while another nomogram developed from the same variables, along with myosteatosis, provided reliable prognostic estimates for their 12-month OS (C-index: 0.69). This underscores the high accuracy and reliability of the survival prediction model in effectively forecasting the survival period and disease progression in unresectable PDAC patients. In the future, the performance of our survival prediction model may be enhanced by integrating more clinical information, CT-based radiomics, and inflammation markers.

Our study limitations included a retrospective design, leading to potential information bias; conducting the study at a single institution, possibly leading to geographical and sample selection biases; heterogeneity in treatment regimens among patients, and limited sample size affecting statistical significance and generalizability of results. Additionally, other unconsidered factors, such as genetic variations, environmental factors, *etc*., may have also impacted our study findings.

## CONCLUSION

In summary, our research has indicated a link among sarcopenia, myosteatosis, and poor prognosis in patients with unresectable PDAC receiving chemotherapy. Early screening, customized treatment strategies, and collaborative efforts among multidisciplinary teams can enhance patient outcomes by identifying and addressing sarcopenia and myosteatosis.

## Figures and Tables

**Table 1:** Patient characteristics and treatment response; comparison of variables by sarcopenia and myosteatosis (n = 150).

-	**Total**	**-**	Sarcopenia	-	**-**	Myosteatosis	-
-	150	Yes (n=57)	No (n=93)	P	Yes(n=39)	No (n=111)	P
Age, years	62.77	64.158	61.925	0.159	63.667	62.46	0.492
Sex(n, %)	-	-	-	-	-	-	-
Male	100(66.67)	41(71.93)	59(63.44)	-	23(58.97)	77(69.37)	-
Female	50(33.33)	16(28.07)	34(36.56)	0.284	16(41.02)	34(30.63)	<0.001
Tumor size, cm	4.14	4.061	4.182	0.668	3.769	4.265	0.109
Tumor size, >3cm(n, %)	107(71.33)	37(64.91)	70(75.27)	0.173	23(58.97)	84(75.68)	0.047
Position(n, %)	-	-	-	-	-	-	-
Head and neck	76(50.67)	32(56.14)	44(47.31)	-	22(56.41)	54(48.65)	-
Body and tail	74(49.33)	25(43.86)	49(52.69)	0.294	17(43.59)	57(51.35)	0.404
Methods of treatment(n, %)	-	-	-	0.719	-	-	0.863
AG	39(26)	17(29.82)	22(23.66)	-	12(30.77)	27(24.32)	-
mFFX	51(34)	18(31.58)	33(35.48)	-	12(30.77)	39(35.14)	-
AG+Immunotherapy	26(17.33)	11(19.30)	15(16.13)	-	7(17.95)	19(17.12)	-
mFFX+Immunotherapy	34(22.67)	11(19.30)	23(24.73)	-	8(20.51)	26(23.42)	-
TNM(n, %)	-	-	-	-	-	-	-
T	-	-	-	0.556	-	-	0.339
1	5(3.33)	1(1.75)	4(4.30)	-	2(5.13)	3(2.70)	-
2	23(15.33)	11(19.30)	12(12.90)	-	9(23.07)	14(12.61)	-
3	21(14)	9(15.79)	12(12.90)	-	4(10.26)	17(15.32)	-
4	101(67.33)	36(63.16)	65(69.89)	-	24(61.54)	77(69.37)	-
N	-	-	-	0.275	-	-	0.632
0	60(40)	25(43.86)	35(37.63)	-	18(46.15)	42(37.84)	-
1	54(36)	16(28.07)	38(40.86)	-	12(30.77)	42(37.84)	-
2	36(24)	16(28.07)	20(21.51)	-	9(23.08)	27(24.32)	-
M	-	-	-	0.163	-	-	0.427
0	58(38.67)	18(31.58)	40(43.01)	-	13(33.33)	45(40.54)	-
1	92(61.33)	39(68.42)	53(56.99)	-	26(66.67)	66(59.46)	-
Stage	-	-	-	0.163	-	-	0.427
3	58(38.67)	18(31.58)	40(43.01)	-	13(33.33)	45(40.54)	-
4	92(61.33)	39(68.42)	53(56.99)	-	26(66.67)	66(59.46)	-
Smoking(n, %)	41(27.33)	18(31.58)	23(24.73)	0.361	10(25.64)	31(27.93)	0.783
Drinking(n, %)	34(22.67)	13(22.81)	21(22.58)	0.974	10(25.64)	24(21.62)	0.606
Co-morbidities (n, %)	-	-	-	-	-	-	-
Solid malignancy	13(8.67)	5(8.77)	8(8.60)	0.971	7(17.95)	6(5.41)	0.017
Diabetes	24(16)13	9(15.79)	15(16.13)	0.956	7(17.95)	17(15.32)	0.7
Hypertension	44(29.33)	10(17.54)	34(36.56)	0.013	12(30.77)	32(28.83)	0.819
Coronary disease	12(8)	7(12.28)	5(5.38)	0.13	5(12.82)	7(6.30)	0.556
Underlying lung diseases	23(15.33)	9(15.79)	14(15.05)	0.903	5(12.82)	18(16.22)	0.613
Hepatitis B Virus	11(7.33)	5(8.77)	6(6.45)	0.597	2(5.13)	9(8.11)	0.539
Laboratory Tests	-	-	-	-	-	-	-
WBC(×10^9/L)	6.45	6.192	6.616	0.24	6.302	6.509	0.606
Lym(×10^9/L)	1.35	1.32	1.375	0.497	1.285	1.378	0.303
Mono(×10^9/L)	0.45	0.443	0.459	0.64	0.438	0.458	0.6
PLT (×10^9/L)	195.17	191.965	197.123	0.647	6.993	65.025	0.1
ALT (U/L)	75.55	77.754	74.196	0.879	92.539	69.578	0.375
TbiL (μmol/L)	42.23	47.826	38.941	0.506	52.203	38.807	0.361
ALB(g/L)	42.48	41.826	42.885	0.202	42.58	42.449	0.887
Cr (μmol/L)	69.6	67.211	71.065	0.216	70.41	69.315	0.751
INR	1.58	2.406	1.074	0.335	3.024	1.073	0.335
CEA(ng/mL)	22.98	25.73	21.29	0.747	28.961	20.875	0.596
CA125(U/mL)	244.97	368.32	169.364	0.185	317.436	219.505	0.519
CA19-9(U/mL)	5214.58	8456.388	3227.661	0.121	3415.051	5846.843	0.415
LMR	3.54	3.457	3.595	0.687	3.487	3.562	0.842
PLR	160.08	163.392	158.048	0.672	159.33	160.341	0.942
Anthropometric variables	-	-	-	-	-	-	-
BMI(kg/m^2^)	21.54	20.804	21.997	0.012	22.033	21.372	0.21
SMI(cm2/m^2^)	40.91	35.253	44.372	<0.001	37.879	41.965	0.005
CSA(cm2)	111.33	98.211	119.37	<0.001	101.886	114.647	0.006
SMD(HU)	43.08	41.035	44.333	0.017	36.333	45.451	<0.001
Best response(n, %)	-	-	-	0.031	-	-	0.142
CR	1(0.67)	1(1.75)	0	-	1(2.56)	0	-
PR	103(68.67)	34(59.65)	69(74.19)	-	24(61.54)	85(76.58)	-
SD	37(24.67)	15(26.32)	22(23.66)	-	12(30.77)	22(19.82)	-
PD	9(6)	7(12.28)	2(2.15)	-	2(5.13)	4(3.60)	-
Objective tumor response	104(69.33)	35(61.4)	69(74.19)	0.099	25(64.1)	85(76.57)	0.130
Disease control	141(94)	50(87.72)	91(97.85)	0.011	37(94.87)	107(96.4)	0.676
Sarcopenia	57(38)	/	/	/	21(53.85)	36(32.43)	0.018
Myosteatosis	39(26)	21(36.84)	18(19.35)	0.018	/	/	/
PFS(months)	10.53	8.15	11.98	0.004	7.97	11.42	0.021
OS(months)	14.76	11.55	16.73	<0.001	11.45	15.93	0.002

**Note:** All data are presented as N (%) unless otherwise indicated. **Abbreviations:**M, metastasis;WBC, white blood cell count, normal range: 4.0-10.0 x10^9/L; Lym, lymphocytes, normal range: 0.8-4.0 x10^9/L;Mono, monocytes, normal range: 0.12-1.00 x10^9/L; PLT, platelet count, normal range: 83-303x 10^9/L;ALT, alanine aminotransferase, normal range: 9-50 U/L; Tbil, total bilirubin, normal range: 0.0-26.0 μmol/L; ALB, albumin, normal range: 40.0-55.0 g/L; Cr, creatinine, normal range: 57-97μmol/L;INR, international normalized ratio, normal range: 0.85-1.15; CEA, carcinoembryonic antigen, normal range: 0.0-5.0 ng/mL; CA125, cancer antigen 125, normal range: 0.0-35.0 U/mL;CA19-9, cancer antigen 19-9, normal range: 0.0-37.0 U/mL; LMR, lymphocyte-to-monocyte ratio, normal range: ≥ 3.0; PLR, platelet-to-lymphocyte ratio, normal range: ≤ 150. CR, complete response; PR, partial response; SD, stable disease; PD, progressive disease. All data are presented as N (%) unless otherwise indicated. Categorical variables were compared using Fisher’s exact test. Ordered variables following normal or non-normal distribution were compared using Student’s t-test or Wilcoxon rank-sum test, respectively. *p value indicates a significant difference.

**Table 2:** Factors associated with PFS and OS based on univariate and multivariate analyses.

-	**PFS**	-	-	-	**OS**	-	-	-
-	**Univariate**	-	**Multivariate**	-	**Univariate**	-	**Multivariate**	-
Parameter	HR(95%CI)	p	HR(95%CI)	p	HR(95%CI)	p	HR(95%CI)	p
Age, years	1.003(0.986-1.021)	0.72	-	-	1.003(0.985-1.021)	0.76	-	-
Sex (male *vs*. female)	0.873(0.604-1.261)	0.468	-	-	0.940(0.650-1.358)	0.74	-	-
Tumor size, cm	0.985(0.887-1.093)	0.774	-	-	0.980(0.884-1.085)	0.693	-	-
Tumor size, >3cm(n, %)	1.048 (0.714-1.538)	0.810	-	-	0.965 (0.659-1.413)	0.856	-	-
Position (Head and neck *vs*. Body and tail	0.985(0.699-1.389)	0.933	-	-	0.820(0.582-1.157)	0.258	-	-
Methods of treatment	/	0.321	-	-	-	0.489	-	-
TNM	-	-	-	-	-	-	-	-
T	/	0.031*	-	-	/	0.085	-	-
N	/	0.037*	-	-	/	0.486	-	-
M	2.107(1.456-3.049)	<0.001*	2.101(1.442-3.063)	<0.001*	2.017(1.399-2.909)	<0.001*	2.235(1.526-3.272)	<0.001*
Stage	2.107(1.456-3.049)	<0.001*	-	-	2.017(1.399-2.909)	0.001*	-	-
Smoking	0.965(0.656-1.420)	0.858	-	-	0.915(0.622-1.347)	0.653	-	-
Drinking	1.099(0.734-1.647)	0.647	-	-	0.999(0.666-1.500)	0.998	-	-
Co-morbidities	-	-	-	-	-	-	-	-
Solid malignancy	0.934(0.503-1.734)	0.829	-	-	0.869(0.468-1.614)	0.657	-	-
Diabetes	0.952(0.591-1.534)	0.839	-	-	0.890(0.553-1.435)	0.633	-	-
Hypertension	0.921(0.631-1.345)	0.671	-	-	0.805(0.551-1.177)	0.264	-	-
Coronary disease	0.81(0.413-1.614)	0.56	-	-	0.689(0.349-1.361)	0.283	-	-
Underlying lung diseases	0.964(0.587-1.524)	0.82	-	-	0.953(0.591-1.536)	0.843	-	-
Hepatitis B Virus	1.163(0.608-2.226)	0.648	-	-	0.893(0.467-1.707)	0.732	-	-
Laboratory Tests	-	-	-	-	-	-	-	-
WBC(×109/L)	0.973(0.891-1.062)	0.535	-	-	0.969(0.886-1.059)	0.484	-	-
Lym(×109/L)	0.538(0.374-0.775)	<0.001*	0.612(0.410-0.913)	0.016*	0.570(0.399-0.814)	0.002*	-	-
Mono(×109/L)	1.222(0.528-2.827)	0.64	-	-	1.390(0.593-3.256)	0.449	-	-
PLT (×109/L)	0.999(0.996-1.001)	0.375	-	-	0.999(0.997-1.002)	0.663	-	-
ALT (U/L)	1.001(1.000-1.002)	0.152	-	-	1.001(1.000-1.002)	0.042*	-	-
TbiL (μmol/L)	1.002(1.000-1.004)	0.108	-	-	1.001(0.999-1.003)	0.295	-	-
ALB(g/L)	1.004(0.972-1.038)	0.804	-	-	0.994(0.962-1.026	0.694	-	-
Cr (μmol/L)	1.002(0.994-1.010)	0.705	-	-	1.001(0.992-1.009)	0.865	-	-
INR	1.005(0.980-1.030)	0.697	-	-	1.003(0.978-1.028)	0.839	-	-
CEA(ng/mL)	1.003(1.001-1.005)	0.008*	1.003(1.001-1.005)	0.010*	1.003(1.000-1.005)	0.017*	-	-
CA125(U/mL)	1.000(1.000-1.000)	0.786	-	-	1.000(1.000-1.000)	0.635	-	-
CA19-9(U/mL)	1.000(1.000-1.000)	0.888	-	-	1.000(1.000-1.000)	0.345	-	-
LMR	0.887(0.805-0.978)	0.016*	-	-	0.891(0.808-0.984)	0.022*	-	-
PLR	1.003(1.000-1.005)	0.025*	-	-	1.003(1.001-1.005)	0.014*	-	-
Anthropometric variables	-	-	-	-	-	-	-	-
BMI(kg/m^2^)	1.058(0.996-1.124)	0.069	-	-	1.024(0.963-1.088)	0.453	-	-
SMI(cm2/m^2^)	0.984(0.962-1.006)	0.152	-	-	0.979(0.958-1.001)	0.066	-	-
CSA(cm2)	0.997(0.990-1.005)	0.466	-	-	0.996(0.989-1.003)	0.217	-	-
SMD(HU)	0.984(0.967-1.002)	0.077	-	-	0.983(0.966-1.001)	0.068	-	-
Best response	-	<0.001*	-	-	/	<0.001*	-	-
ORR	1.970(1.372-2.828)	<0.001*	1.682(1.114-2.541)	0.013*	2.004(1.396-2.877)	<0.001*	1.660(1.100-2.506)	0.016*
DCR	8.591(4.045-18.246)	<0.001*	5.130(2.217-11.869)	<0.001*	2.851(1.435-5.663)	0.003*	2.343(1.059-5.183)	0.036*
Sarcopenia	1.714(1.203-2.442)	0.003*	1.699(1.165-2.478)	0.006*	2.036(1.421-2.918)	<0.001*	2.023(1.394-2.934)	<0.001*
Myosteatosis	1.654(1.123-2.435)	0.011*	-	-	1.811(1.230-2.666)	0.003*	1.735(1.154-2.608)	0.008**

**Abbreviations:** HR, hazard ratio; CI, confidence interval; ORR, objective response rate; DCR, disease control rate. *p value indicates a significant difference.

## Data Availability

All data generated or analyzed during this study are included in this published article.

## LIST OF ABBREVIATIONS

CT: Computed Tomography
PDAC: Pancreatic Ductal Adenocarcinoma
PFS: Progression-free Survival
OS: Overall Survival
mFFX: modified Folfirinox
HCC: Hepatocellular Carcinom
LMR: Lymphocyte-to-monocyte Ratio
PLR: Platelet-to-lymphocyte Ratio

## STANDARDS OF REPORTING

STROBE guidelines were followed.
